# A Rare Presentation of Hepatocellular Carcinoma: Adrenal Metastasis Mimicking Primary Adrenal Malignancy

**DOI:** 10.7759/cureus.83344

**Published:** 2025-05-02

**Authors:** Ryan D Muchard, Erin G Park, Carson Woodward, Christopher D Johnson

**Affiliations:** 1 Medicine, Alabama College of Osteopathic Medicine, Dothan, USA; 2 General Surgery, Advent Health Tampa, Tampa, USA; 3 General Surgery, Ascension Sacred Heart Bay, Panama City, USA

**Keywords:** adrenal glands, adrenal malignancy, hepato cellular carcinoma, robotic surgical procedures, tumor removal

## Abstract

Hepatocellular carcinoma (HCC), a primary liver malignancy, often metastasizes to extrahepatic organs, including the adrenal glands. Differentiating metastatic HCC from primary adrenal tumors is diagnostically challenging, particularly in patients with chronic liver disease, and underscores the significance of this rare case.

A 74-year-old African American male with a history of hepatitis C and hepatic fibrosis treated eight years prior, presented with significant weight loss, left upper quadrant pain, and an 8 cm positron emission tomography (PET)-avid, standard uptake value (SUV) 6.2 left adrenal mass. Imaging and biochemical evaluations suggested a non-functional adrenal tumor, initially favoring a primary adrenal malignancy. Surgical resection revealed high-grade hepatoid carcinoma on histopathology, and immunohistochemistry indicated either a primary adrenal tumor or metastatic HCC. Postoperative biopsy of a concurrent hepatic lesion confirmed HCC, establishing the adrenal mass as a rare case of metastatic HCC with the adrenal gland as the dominant presentation.

This case underscores the diagnostic challenges in differentiating between primary adrenal tumors and metastatic HCC in patients with chronic liver disease. A multidisciplinary approach incorporating imaging, biopsy, histopathology, and immunohistochemistry is essential for accurate diagnosis. This rare presentation emphasizes the importance of considering metastatic HCC in adrenal masses associated with liver pathology, enhancing clinical awareness and diagnostic precision.

## Introduction

Hepatocellular carcinoma (HCC) is known for its propensity to metastasize to various organs, including the lungs, bones, lymph nodes, and adrenal glands. Adrenal metastases from HCC are not relatively common, being identified in approximately 8.4% of autopsy cases, making the adrenal gland a less frequent site of metastasis, with the lungs being most common [1,2]. In cases of adrenal involvement, distinguishing between primary adrenal tumors and secondary metastases can be challenging, requiring detailed clinical and pathological evaluation. This case report discusses a 74-year-old African American male with a history of hepatitis C and hepatic fibrosis treated eight years prior, presenting with a Positron Emission Tomography (PET) avid 8 cm left adrenal mass. The patient's clinical presentation, including significant weight loss and left upper quadrant pain, alongside imaging findings, raised concerns for a primary adrenal malignancy. However, given his history of Hepatitis C, the differential diagnosis must also consider metastatic HCC, as chronic liver disease significantly increases the risk of primary liver malignancies and their subsequent metastases [3-5]. This case underscores the importance of a thorough diagnostic evaluation, including imaging, biopsy, biochemical workup, histopathological and immunohistochemical analyses such as alpha-fetoprotein (AFP) positivity, to differentiate between primary adrenal tumors and metastatic lesions, particularly in patients with a history of chronic liver disease.

## Case presentation

A 74-year-old African American male with a history of a PET-avid 8 cm left adrenal mass concerning for primary malignancy presented for preoperative evaluation. The patient initially experienced left upper quadrant pain six months ago, along with a significant 30-pound weight loss over that period. Cross-sectional imaging and PET scans revealed the adrenal lesion. A biochemical workup with low-dose dexamethasone suppression test, 24-hour urine cortisol, metanephrines, aldosterone, vanillylmandelic acid, and catecholamines confirmed it to be non-functional, ruling out a cortisol, aldosterone, or catecholamine-producing tumor. Despite recent recovery from coronavirus disease 2019 (COVID-19), he remained asymptomatic with no new complaints. 

The patient has a significant medical history, including well-controlled diabetes and hypertension, managed with a single antihypertensive medication. Eight years ago, he was successfully treated for Hepatitis C, with negative Hepatitis C RNA. Hepatic fibrosis or cirrhosis remains a concern, potentially influencing the differential diagnosis by raising the likelihood of metastatic hepatocellular carcinoma (HCC). Additionally, he has a past history of a transient ischemic attack (TIA) and reports memory loss.

Vital signs on preoperative evaluation were noncontributory, revealing blood pressure of 155/83 mmHg, heart rate of 76 beats per minute, respiratory rate of 18 breaths per minute, and temperature of 98° F. Further preoperative evaluation revealed hypertension, mild anemia, and elevated liver enzymes. Key laboratory values and vital signs are summarized in Table 1.

**Table 1:** Laboratory results: chemistry, liver enzymes, complete blood count

Laboratory Results			
Test	Result	Reference	Units
Sodium	140	136-145	mEq/L
Potassium	4.5	3.5-5	mEq/L
Chloride	107	95-105	mEq/L
CO_^2^_	27	23-30	mEq/L
Blood Urea Nitrogen	18	7-21	mg/dL
Creatine	1	0.6-1.2	mg/dL
Glucose	80	74-99	mg/dL
Estimated Glomerular Filtration Rate	79	>90	mL/min/1.73 m^2^
Aspartate Transaminase (AST)	120	10-40	U/L
Alanine Transaminase (ALT)	106	7-56	U/L
Alkaline Phosphatase (ALP)	122	44-147	U/L
Bilirubin Total	0.7	0.1-1.2	mg/dL
White Blood Cell Count	9,000	4,000- 11,000	/mm^3^
Hemoglobin	12.6	13.5-17.5	g/dL
Hematocrit	38	41–53	%
Platelet Count	167,000	150,000–400,000	/mm^3^

**Figure 1:**
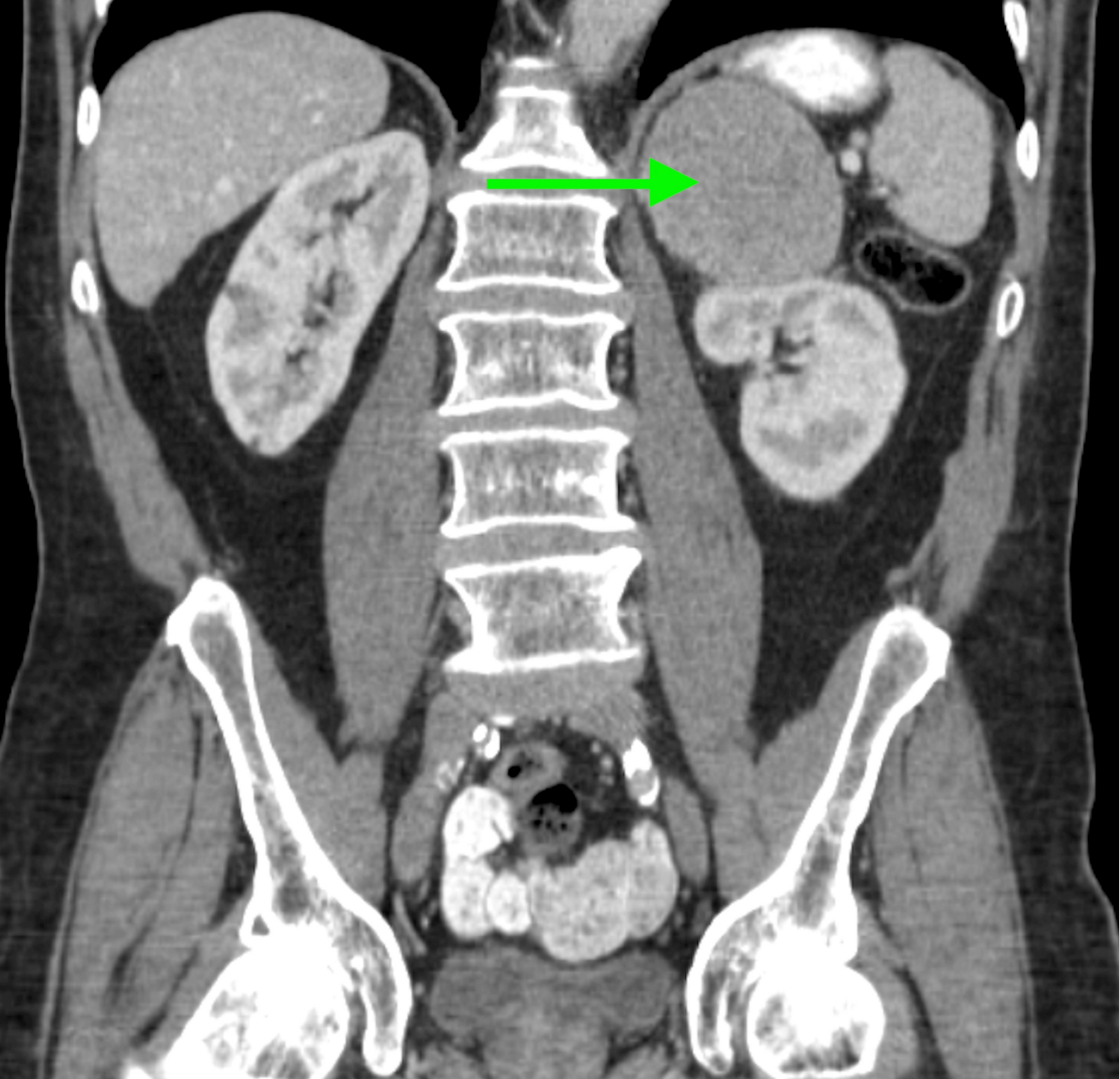
Non-contrast CT scan of the chest and abdomen Green arrow points to the 8 × 11 cm mass in the left upper quadrant, originating from the adrenal gland.

**Figure 2:**
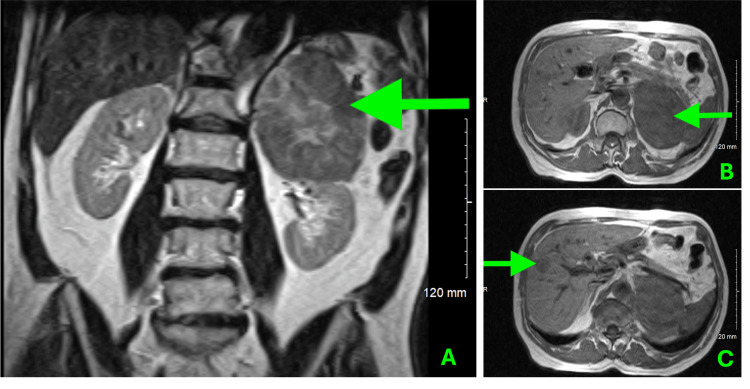
MRI Imaging A. T1-weighted coronal MRI slice shows a large mass in the left adrenal gland indicated by the green arrow. B. Axial MRI slice demonstrates the tumor's full width, green arrow indicating the adrenal mass. C. The green arrow indicates the 2.8 cm hepatic lesion observed in the right lateral segment of the liver.

Initial diagnostic imaging included a Computed Tomography (CT) scan of the chest and abdomen without contrast, which showed an 8 × 11 cm mass in the left upper quadrant abutting the left kidney and adrenal in origin (Figure 1). Subsequent Magnetic Resonance Imaging (MRI) of the abdomen with and without contrast showed a large heterogeneous enhancing mass within the superior left retroperitoneum, inseparable from the superior lobe of the left kidney. At this point in the diagnostic process, findings were favorable to represent a primary left adrenocortical carcinoma. An incidental indeterminate 2.8 cm enhancing heterogeneous mass within the right lateral liver was noted during imaging and is concerning for possible metastasis from the adrenal mass versus an unrelated lesion (Figure 2). No further workup or biopsy of the liver lesion was pursued at this time, as metastatic HCC was not a primary consideration in the differential diagnosis due to its atypical presentation.

**Figure 3:**
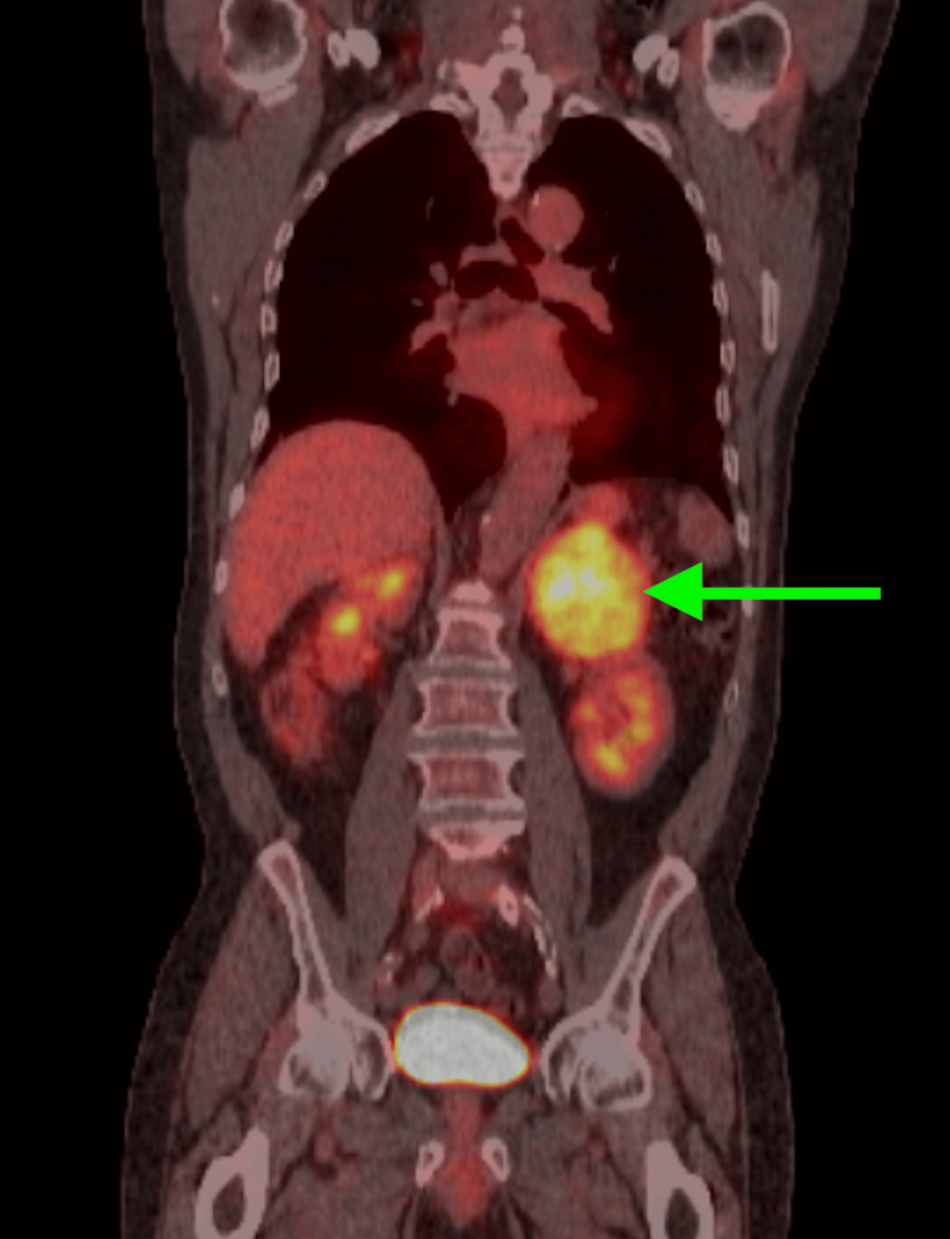
PET scan The green arrow is showing increased radiopharmaceutical uptake in the left adrenal gland.

PET scan of the head, neck, chest, abdomen, and pelvis following intravenous injection of 12.4 ML of F-18 deoxyglucose (FDG) shows increased FDG concentration in the left upper abdomen associated with a markedly enlarged left adrenal gland. The maximal axial diameter of the left adrenal gland is 85.4 mm, and the calculated standard uptake value is 6.2. Normal physiologic distribution of the radiopharmaceutical is identified in the hepatic parenchyma (Including the 2.8 hepatic mass), splenic parenchyma, renal units, urinary bladder, and visualized intestinal tract. The abnormal examination was indicative of a malignant-viable neoplasm in the adrenal gland. Increased radiopharmaceutical concentration identified in the left adrenal gland fulfilled quantitative criteria for malignant transformation (Figure 3). No other qualitatively significant abnormalities were noted. The normal standard uptake value of the hepatic lesion and its non-visualization on the PET scan added diagnostic complexity, leading the clinical team to consider the adrenal mass as a primary malignancy initially. Only after a postoperative biopsy of the hepatic lesion was the diagnosis of HCC confirmed, establishing the adrenal mass as a metastatic lesion rather than a primary adrenal tumor. 

**Figure 4:**
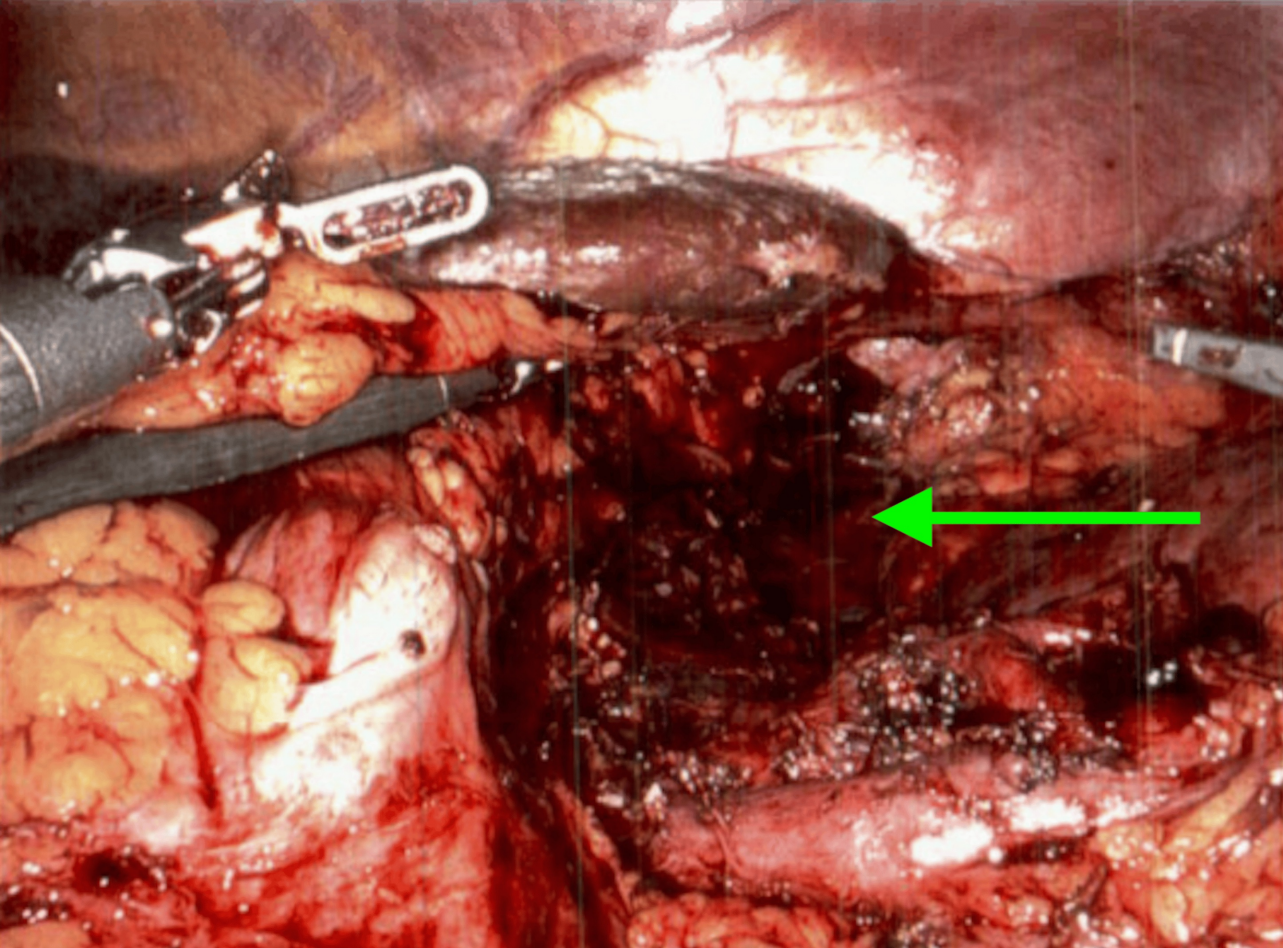
Successful left adrenalectomy Intraoperative imaging shows the cavity left after the mass excision indicated by green arrow.

The patient underwent a robotic-assisted left adrenalectomy for a large, approximately 14 cm adrenal mass with adhesion but not invasion into the left renal vein, with displacement of the left kidney inferiorly. Intraoperative findings revealed a large adrenal mass with omental adhesions to the anterior abdominal wall and left colon. The colon and splenic flexure were meticulously mobilized, preserving the spleen and pancreas. The mass displaced the renal vein inferiorly, requiring careful dissection around the renal vasculature. A portion of the mass adhered to a loop of the renal artery, which was successfully dissected (Figure 4). The mass was removed through an extended midline incision without injury to surrounding structures and sent to pathology. On final inspection, no pathologic lymph nodes were identified, and no injuries to the colon, pancreas, spleen, or kidneys were observed. The procedure was completed without intraoperative complications, with an estimated blood loss of 50 cc. A drain was placed, and the patient was transferred to recovery in stable condition. The procedure was completed by a general surgeon and assisted by a urologist while dissecting the renal vasculature. 

The excised left adrenal mass measured 14 cm and weighed 478 grams, with focal extension to the disrupted capsular surface. Per the pathology report, Histopathology revealed a high-grade hepatoid carcinoma involving the left adrenal gland, with the differential diagnosis including primary adrenal carcinoma versus metastatic high-grade hepatocellular carcinoma. Clinical correlation was advised for further clarification. Immunohistochemistry showed positivity for anti-cytokeratin (CAM5.2), hepatocyte-specific antibody (HSA), and focal CD56, while markers such as alpha-fetoprotein (AFP), vimentin, and synaptophysin were negative. A high Ki-67 proliferation rate of 60% was noted, indicating aggressive tumor behavior (Table 2). No lymphatic or vascular invasion was identified. The absence of AFP, a marker commonly elevated in hepatocellular carcinoma, highlights the lack of definitive tumor-specific markers for hepatocellular origin and adds complexity to the differentiation between metastatic HCC and primary adrenal carcinoma. Although the findings suggest a primary adrenal tumor, the patient's history of hepatitis C underscores the necessity of integrating clinical, radiologic, and histopathologic data to resolve the diagnostic uncertainty. Biopsy of the liver lesion was now pivotal in confirming the diagnosis of metastatic HCC, emphasizing the critical role of histopathological confirmation in guiding management in such complex cases.

**Table 2:** Tumor marker analysis for adrenal mass CAM5.2: Anti-cytokeratin; HSA: Hepatocyte specific antibody; AFP: Alpha-fetoprotein; AE1/AE2: Pan-cytokeratin; GATA3: GATA binding protein 3; PAX8: Paired box gene 8; CEA: Carcinoembryonic antigen.

Tumor marker analysis for adrenal mass	
Marker	Result
CAM5.2	Positive
HSA	Positive
CD56	Focally Positive
Ki-67	High (60%)
AFP	Negative
AE1/AE3	Negative
CD45	Negative
Vimentin	Negative
Inhibin	Negative
Mart-1	Negative
S100	Negative
p63	Negative
Synaptophysin	Negative
Chromogranin	Negative
GATA3	Negative
PAX8	Negative
Calretinin	Negative
MOC-31	Negative
BEREP4	Negative
CEA	Negative

Postoperatively, the patient was placed on a multimodal pain management regimen, which provided effective pain control. His diet was successfully reintroduced and progressively advanced without complications. Due to slow progress in physical and occupational therapy, inpatient rehabilitation services were consulted. The patient was eventually discharged to a rehabilitation facility to continue his recovery. 

**Figure 5:**
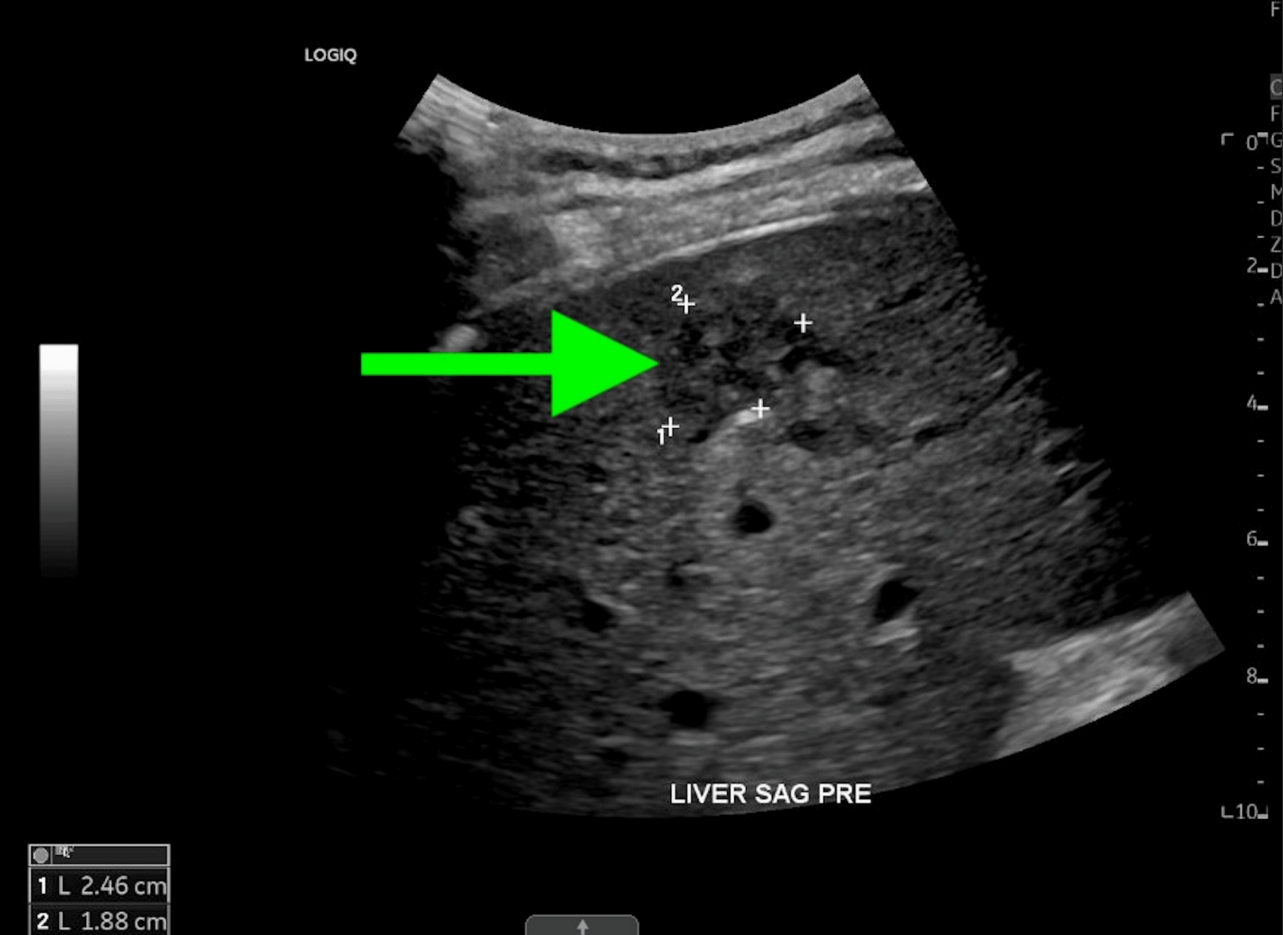
Ultrasound imaging of the liver The green arrow is demonstrating the hepatic mass during Interventional radiology core needle biopsy. Sonographic evaluation of the right hepatic lobe demonstrates a mixed echogenic mass.

Approximately four weeks postoperative, due to the patient's delayed recovery and placement in a rehabilitation facility, an ultrasound-guided core needle biopsy of the right hepatic lobe 2.8 cm mass was performed (Figure 5). The patient tolerated the procedure well without immediate complications. The pathology report indicated a highly AFP-positive tumor consistent with HCC. Serum AFP level of 82.5 ng/mL was recorded at this time. (normal: less than 10 ng/mL). A multidisciplinary discussion determined that the adrenal mass was metastatic HCC, linked to the patient's chronic liver disease, including Hepatitis C and potential hepatic fibrosis, which are well-documented risk factors for HCC and its metastatic spread. Following resection, the patient was initiated on atezolizumab and bevacizumab, completing seven cycles to date. He continues to tolerate the treatment well, with follow-up imaging via CT chest and abdominal MRI demonstrating stable disease and no evidence of new metastases.

## Discussion

Hepatoid carcinoma and hepatocellular carcinoma (HCC) are distinct entities that can present diagnostic challenges due to their morphological similarities. Hepatoid carcinoma is an extrahepatic adenocarcinoma that exhibits features resembling HCC, including the production of alpha-fetoprotein (AFP) and histological patterns [6-8]. Hepatoid carcinoma can arise in various organs, such as the stomach, lung, pancreas, and ovary. It is characterized by the presence of hepatoid cells, which are morphologically similar to hepatocytes, and often shows positive immunohistochemical staining for markers like AFP, HepPar1, and cytokeratins CK8 and CK18. However, it may also express markers not typically seen in HCC, such as CK19 and CK20, which can aid in differentiation [6-8]. Hepatocellular carcinoma (HCC), on the other hand, is a primary liver malignancy commonly associated with risk factors such as chronic hepatitis B or C infection, alcohol-related liver disease, and non-alcoholic steatohepatitis. HCC typically shows positive staining for HepPar1, arginase-1, and glypican-3 but lacks expression of markers like CK19 and CK20 [6,9-10].

To distinguish between these two entities, a comprehensive approach involving clinical history, imaging studies, and a detailed immunohistochemical panel is essential. For instance, hepatoid carcinoma often presents with elevated AFP levels. It can be found in patients without liver disease risk factors, whereas HCC is usually associated with underlying liver pathology and specific risk factors [6-8]. The patient's history of successfully treated hepatitis C with concerns for hepatic fibrosis or cirrhosis significantly impacts the diagnosis of the PET-avid 8 cm left adrenal mass. Chronic hepatitis C infection and subsequent hepatic fibrosis or cirrhosis increase the risk of hepatocellular carcinoma (HCC) [11]. Given this background, the possibility of metastatic HCC must be considered, as HCC can metastasize to the adrenal glands.

The American Gastroenterological Association guidelines emphasize that in patients with underlying liver disease, HCC should always be at the top of the differential diagnosis when evaluating solid liver lesions [11]. This is particularly relevant in this case, where the patient has a history of hepatitis C and potential cirrhosis, both of which are significant risk factors for HCC development.

Additionally, the presence of an indeterminate hepatic lesion on MRI further raises the suspicion of metastatic disease, potentially from an undiagnosed primary HCC. Therefore, while the adrenal mass could represent a primary adrenal malignancy, the patient's hepatic history necessitates a thorough evaluation for metastatic HCC, including further imaging and biopsy of the hepatic lesion, which confirms the diagnosis of HCC. In this case, both MRI and PET-CT scans were pivotal in evaluating the adrenal and hepatic masses. The MRI indicated an indeterminate hepatic lesion with characteristics that raised suspicion of malignancy, while the PET scan demonstrated FDG uptake only in the adrenal gland and not the liver, consistent with a primary malignant process. These imaging modalities collectively reinforced the need for further diagnostic measures, including biopsy.

In summary, the patient's history of hepatitis C with concerns for hepatic fibrosis or cirrhosis increases the likelihood of metastatic hepatocellular carcinoma as a diagnosis for the PET-avid adrenal mass [11].

## Conclusions

This case underscores the diagnostic challenges of distinguishing primary adrenal malignancies from metastatic hepatocellular carcinoma (HCC), particularly in patients with chronic liver disease. Comprehensive evaluation through imaging, histopathology, and immunohistochemistry is critical for accurate diagnosis. The patient's history of hepatitis C and hepatic fibrosis highlights the need for vigilance in similar scenarios. This case emphasizes the value of a multidisciplinary approach in managing complex adrenal masses, ensuring precise diagnosis and optimal care. The findings also contribute to the broader understanding of adrenal metastases in HCC, demonstrating how chronic liver disease and its sequelae can obscure the clinical picture, complicating timely diagnosis and management. By reporting this rare presentation, this case highlights the importance of integrating patient history with advanced diagnostic tools to guide effective therapeutic strategies.
